# Gastroesophageal reflux disease incidence among male patients with irritable bowel syndrome: A single‐center cross‐sectional study in southern Iran

**DOI:** 10.1002/jgh3.12867

**Published:** 2023-01-27

**Authors:** Fereshteh Gholamnezhad, Ahmad Qeisari, Reza Shahriarirad

**Affiliations:** ^1^ School of Medicine Islamic Azad University, Kazeroun Branch Kazeroun Iran; ^2^ Student Research Committee, School of Medicine Shiraz University of Medical Sciences Shiraz Iran; ^3^ Thoracic and Vascular Surgery Research Center Shiraz University of Medical Science Shiraz Iran

**Keywords:** gastrointestinal reflux disease, Iran, irritable bowel syndrome, male

## Abstract

**Background and Aim:**

Gastrointestinal reflux disease (GERD) and irritable bowel syndrome (IBS) are among the most common gastrointestinal disorders in which the overlap of these diseases and their syndromes has been frequently reported. In this study, we aimed to evaluate GERD incidence among IBS patients and the related risk factors.

**Methods:**

Male patients aged 18–60 years with an impression of IBS and referred to the gastrointestinal clinic from March 2019 to 2020 in Shiraz, Iran, were included in this study.

**Results:**

Among the 163 enrolled patients with an average age of 31.53 ± 9.38 years, 64 (39.3%) were diagnosed with GERD. Based on statistical analysis, there was a significant association between GERD and the IBS patients' age (*P* = 0.006), smoking (*P* = 0.011), and alcohol consumption (*P* = 0.043). Also, GERD among IBS patients was significantly associated with the type of IBS (*P* < 0.001), with IBS‐D having the lowest incidence (19.4%) and IBS‐M the highest incidence of GERD (66.7%). Based on multivariate analysis, smoking had a reverse and significant correlation with lower incidence of GERD (OR = −1.364; *P* = 0.002).

**Conclusion:**

Our results demonstrate that among male IBS patients, younger age, smoking, and alcohol consumption were among the risk factors for GERD. These findings may provide further insight into the best approach to treating these diseases.

## Graphical abstract

GERD and IBS are among the most common gastrointestinal disorders in which the overlapping of these diseases and their syndromes has been frequently reported. Our results demonstrated that among male IBS patients, younger age, smoking, and alcohol were among the risk factors for GERD. Improvements and changes in the clinicians and scientists point of view regarding GERD and IBS could provide ways to improve how these disorders are managed.

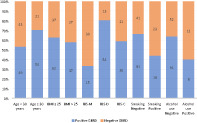

## Introduction

Gastrointestinal reflux disease (GERD) and irritable bowel syndrome (IBS) are among the most common diseases in humans.^1^, ^2^ IBS is a chronic functional disorder of the gastrointestinal system with an increasing trend among the population, in which the prevalence of 9.2–32.5% has been reported among adults.^1^, ^3^ The disease is characterized by abdominal pain and altered bowel habit, with predominantly constipation (IBS‐C), diarrhea (IBS‐D), or both (IBS‐M).^1^, ^4^ GERD is the most common disease of the esophagus, whose hallmarks include heartburn and acid regurgitation.^5^ Its most crucial complication includes esophagitis and Barrett metaplasia, which occurs in approximately 7–15% of patients and increases the risk of progressing into esophageal adenocarcinoma.^6^, ^7^

Reflux symptoms persist in almost 75% of patients for at least 10 years, its symptoms in IBS patients being usually chronic. Therefore, it is not surprising that IBS and GERD overlap in clinical presentations.^8^ In a systematic review, IBS has been reported in 42% of GERD patients.^9^ Furthermore, Some studies have reported that the overlap occurs more among males.^10^ Male sex is associated with increased signs of reflux and also a higher occurrence of esophagitis.^11^ GERD among male patients appears to have more extreme manifestations marked by erosive esophagitis, ulcer of the esophagus, or strictures.^12^, ^13^, ^14^ The reason why males are more vulnerable to getting GERD and IBS together is not known; therefore, further investigation into the potential cause of this gender difference is warranted.

Recent reports by Asian researchers indicate that IBS prevalence is high and equal to that in Western cultures.^15^, ^16^ In a study by Massarrat *et al*.,^17^ male Iranian pastoral nomads of Turkish descent from the south and industrial workers from the north were compared for the prevalence of IBS. Although the nomads consumed less fruits and vegetables, it was believed that their constant travel was responsible for their lower frequency of constipation than that of Western people. Factory employees' arduous physical labor had the same result. On the other hand, factors such as smoking and alcohol use are more prevalent among the male population in Iran,^18^ which can be correlated with both GERD and IBS.^19^, ^20^

Therefore, in this study, we aimed to evaluate GERD's prevalence among male IBS patients in southern Iran. Also, we sought to evaluate the associated risk factors for GERD among IBS patients.

## Methods

### Study design

In this cross‐sectional descriptive study, patients being referred to the Moslemin clinic from March 2019 to March 2020 (1 year) with an IBS impression were included. This clinic is located in Muslemin hospital and among the main centers in Shiraz, Iran. This center is among the most visited referral centers in southern Iran and provides primary health care to the whole population. Subjects were recruited in a consecutive manner from among patients who either visited our center based on their initial symptoms or were referred to us from tertiary and other centers for better evaluation or endoscopic examination. Patients were informed of the aim of the study, and written informed consents were obtained from them. In case a patient expressed unwillingness to participate, the next consecutive patient was recruited until the desired sample size was achieved.

The inclusion criteria consisted of male patients aged 18–60 years and a diagnosis of IBS who were willing to participate in our research and understood the study's content and the questionnaire. The exclusion criteria consisted of age under 18 or above 60 years, with underlying esophageal movement disorders such as achalasia, scleroderma, diffuse esophageal spasm, or lower esophageal sphincter hypotension, inflammatory bowel diseases such as Crohn's and ulcerative colitis, electrolyte disorders, esophageal diverticulum, cancer of the upper gastrointestinal system, esophageal varices, history of intestinal obstruction or gastrointestinal surgery, or any contraindications regarding endoscopy such as severe cardiovascular diseases or respiratory distress.

### Disease diagnosis

For IBS diagnosis, we used the ROME IV criteria, including abdominal pain or discomfort for 12 or more weeks in the past year, although not necessarily consistent, which fulfilled at least two of the three criteria of “Relieved with defecation,” and/or “Onset associated with a change in stool frequency,” and/or “Onset associated with a change in form (appearance) of stool.” Additionally, the GERD hallmark includes heartburn and acid regurgitation based on GERQ.^21^

After diagnosing IBS by a single specialized physician, the patients were included in our study and evaluated for GERD features; then, demographic information of the patients such as age, sex, BMI, and GERD features was collected.

### Statistical analysis

The sample size was calculated based on a study by Lovell and Ford, by reporting a prevalence of GERD in IBS as 42.0% and considering α = 0.05 and confidence level = 80%; a final sample size of 158 patients was arrived at.^9^ The extracted data were analyzed using SPSS version 22.0 (SPSS Inc., Chicago, IL, USA). The data were presented either as descriptive or based on a chi‐square test for evaluating categorical data. Also, statistically significant variables were included in the multivariable logistic regression analysis to adjust for confounding factors. A *P*‐value <0.05 was considered significant association.

### Ethical approval

The study was approved by the Research Ethics Committees of Islamic Azad University‐ Kazerun Branch (Ethical code: IR.IAU.KAU.REC.1399.020) and conducted in compliance with the relevant guidelines and regulations and the Declaration of Helsinki. Written informed consent was obtained from all patients to participate in this study. Patients were free to leave the study at any time. Also, the confidentiality of the patient information was ensured by the researcher.

## Results

In the present study, 163 male patients diagnosed with IBS were enrolled for 1 year. All patients entered the study voluntarily. The participants' age ranged from 18 to 60 years (mean age: 31.53; SD: 9.38). Among the patients, 64 (39.3%) were diagnosed with GERD. Table 1 shows the characteristics of the patients in our study and their association with GERD. Based on the patients′ social history, 23/64 (35.9%) patients with GERD were smokers and 12/64 (18.8%) were alcohol users.

**Table 1:** Demographic and clinical features of male patients with irritable bowel syndrome and its association with gastrointestinal reflux disease in southern Iran

		GERD		
Variable	Total	Yes, *n =* 64	No, *n* = 99	*P*‐value
Mean age ± SD	31.53 ± 9.38	29.82 ± 9.7	32.64 ± 9.05	0.006*
Age group (%)				
<30	92 (56.4)	43 (46.7)	49 (53.3)	0.076
30–39	40 (24.5)	9 (22.5)	31 (77.5)	
40–49	21 (12.9)	8 (38.1)	13 (61.9)	
≥50	10 (6.1)	4 (40)	6 (60)	
Average BMI ± SD	25.05 ± 3.52	25.16 ± 3.24	24.97 ± 3.7	0.531
BMI group (%)				
<25	99 (60.7)	37 (37.4)	62 (62.6)	0.539
≥25	64 (39.3)	27 (42.2)	37 (57.8)	
Social history (%)				
Smoking	41 (25.2)	23 (56.1)	18 (43.9)	0.011*
Alcohol	20 (12.3)	12 (60)	8 (40)	0.043*
Type of IBS (%)				
Mixed	45 (27.6)	30 (66.7)	15 (33.3)	<0.001*
Diarrhea	67 (41.1)	13 (19.4)	54 (80.6)	
Constipation	51 (31.3)	21 (41.2)	30 (58.8)	

* Indicates significant association.

BMI, body mass index; GERD, gastrointestinal reflux disease; IBS, irritable bowel syndrome; SD, standard deviation.

On the basis of statistical analysis, there was a significant association between age and incidence of GERD among IBS patients in our study (*P* = 0.006). Although no significant association was found among the age groups and GERD (*P* = 0.058), by classifying the patients based on <30 years *versus* ≥30, a significant association was found, with patients <30 years having a higher incidence of GERD than patients ≥30 years (67.2% *vs* 32.8%, *P* = 0.035). Figure S1, Supporting information shows the incidence of GERD based on age and BMI groups, social history, and type of IBS.

Based on our results, there was no significant association between the incidence of GERD among IBS patients and BMI or BMI groups (*P* > 0.05). Based on the patients′ social history, a significant and higher association was observed between GERD in IBS patients and smoking (*P* = 0.011) and alcohol consumption (*P* = 0.043).

Also, GERD among IBS patients was significantly associated with the type of IBS (*P* < 0.001), with IBS‐D having the lowest incidence (19.4%) and IBS‐M the highest incidence of GERD (66.7%).

Additionally, we performed multivariate analysis to obtain the confounding factors related to the incidence of GERD among IBS patients; the results are reported in Table 2. As can be seen, although age had a reverse correlation with higher prevalence of GERD, there was no statistical significance (OR: –0.030; *P* = 0.171). However, smoking had a direct and significant correlation with lower incidence of GERD (OR = −1.364; *P* = 0.002). It is also seen that the type of IBS has a significant association with GERD, with type M having a lower correlation compared to type D but a higher correlation compared to type C (Table 2).

**Table 2:** Multivariate regression analysis of risk factors for gastrointestinal reflux disease among male patients with inflammatory bowel syndrome (*N* = 163)

Variable	OR; 95% CI	*P*‐value*
Higher age	−0.028 (0.931–1.014)	0.194
Higher BMI	0.041 (0.936–1.159)	0.452
IBS type^†^		
M (ref.)	—	**<0.001**
D	1.054 (1.183–6.953)	**0.020**
C	–1.265 (0.112–0.714)	**0.008**
Smoker (positive/negative)	–1.361 (0.106–0.619)	**0.002**
Alcohol use (positive/negative)	–0.684 (0.168–1.515)	0.223

* Values in bold indicate significant correlation.

^†^ IBS types are compared to the reference variable (Type M).

BMI, body mass index; CI, confidence interval; OR, odds ratio.

## Discussion

IBS and its comorbidities have recently been the subject of comprehensive study. However, the exact pathogenesis of GERD and IBS is still unknown, and many surveys have suggested the increased prevalence of GERD in IBS and vice versa.^8^, ^22^, ^23^, ^24^ in this study, we aimed to evaluate the incidence of GERD among IBS patients by limiting it to male population for considering the related risk factors among this group. Our results showed a prevalence of 39.3% GERD among male IBS patients with age <30, smoking, and alcohol consumption as the main risk factors.

Our data are in accordance with those in a review by Nastaskin *et al*.,^25^ which reported an overlap of 39% between IBS and GERD. They also noted that the diseases together tend to cover a degree and rate that is greater than their incidence in the population. Furthermore, their data show that if one of the disorders is omitted, the other is less prevalent in the population, which implies a possible causal association.^25^

Although most studies have focused on the gender difference between GERD and IBS, IBS and functional GI conditions are more common in women than in men.^26^ Female‐to‐male ratios of 2–3:1 were recorded in most of the studies.^27^ However, GERD among males appears to have more extreme manifestations.^12^, ^13^, ^28^, ^29^ Also, a higher positive association between GERD and IBS was found to exist predominantly in male subjects in previous studies.^10^ Among the main reasons, one could name the associated risk factors such as alcohol consumption and smoking, which are more frequent among the males, which have also been demonstrated to be risk factors for GERD based on our results. Increasing public knowledge of such risk factors is essential in controlling the incidence of these diseases.

There are many variations regarding the significance of the variables and GERD–IBS overlap among the studies, which justifies the need for evaluating these variables more deeply and extensively and among different populations. Based on our results, younger participants are more vulnerable to both diseases. This finding is similar to that of a study by Cheung *et al*.^10^ In younger subjects, the prevalence of IBS eventually decreases with age.^30^, ^31^ However, some studies have shown that increasing age was not associated with IBS–GERD overlap.^32^ Studies have reported that smoking is a risk factor for GERD^33^; however, its role in IBS is still controversial and also subtype‐based.^34^, ^35^ However, some studies have failed to find any significant association between behavioral factors, such as smoking or alcohol consumption, and IBS–GERD overlap.^32^ However, studies have also reported that functional heartburn has more overlap with IBS than GERD. Since we could not distinguish true GERD from other functional gastrointestinal disorders without a gold standard test, further studies with a more accurate focus in this regard is necessary.^19^

GERD–IBS overlap was significantly lower among IBS‐D patients (19.4%), while IBS‐M patients had the highest GERD incidence (66.7%). IBS‐M type patients are more frequent in our study population, which is in line with previous reports.^4^ In a study by Talley *et al*.,^36^ overlap with GERD‐related symptoms was found in 32.9% of patients with predominant IBS‐C and 40.9% of patients with predominant IBS‐D. The exact mechanism for this association is still not clear; however, IBS‐C is associated with a higher frequency of bloating and early satiety, which possibly represents fundamental pathophysiological pathways distinct from those in IBS‐D.^36^ Brain–gut interaction dysregulation has been proposed as a vital factor in the pathogenesis of IBS.^37^ Especially, IBS‐D has been related with autonomic nervous dysfunction but independent of psychological disturbance.^37^ However, the exact mechanism involved in the overlap of GERD and the subtypes of IBS is still unclear and warrants further investigations.

Based on our results, there was no significant association between BMI and the overlap of GERD and IBS. Our data contrasts with those of a study by Jung *et al*.^32^ reporting that in only IBS but not GERD was a higher BMI a predictor of increasing IBS–GERD overlap. BMI has also been reported a risk factor for GERD,^38^, ^39^ while its role in IBS is controversial.^40^ Increased somatization relative to GERD alone was another independent risk factor for the IBS–GERD overlap.

There is evidence that social stressors may promote gastroesophageal reflux and IBS, and somatization can be a marker of these conditions.^41^, ^42^ Psychological disorders such as anxiety and depression have been reported to influence these two conditions. In a study by Rathi *et al*.^41^ among 100 GERD cases, 56 were found to have psychiatric morbidity in the form of either anxiety or depression, which was not associated with the patients′ demographic and social factors. Dissatisfaction with the body image has been reported to influence depression in GERD.^43^ A study in China found higher anxiety and depression scores in non‐erosive reflux disease compared to reflux esophagitis.^44^ This could indicate that patients with reflux esophagitis are generally less sensitive to pain due to mucosal changes or that patients with non‐erosive reflux disease may perceive GERD symptoms more strongly due to their anxiety or depression. To fully understand this difference, the literature on reliable physiological studies must be examined, but there is strong support for the hypothesis that acid reflux and psychosocial stress are responsible for the higher prevalence of depression and anxiety in these patients.^41^

Among the limitations of our study is that we included only male patients. We hypothesized that, since biological markers, mental factors (such as stress and anxiety levels), and also social settings and behavioral pattern differences among the male and female population in Iran could not be evaluated in detail in our study, which could each effect the prevalence and incidence of the disease;^45^, ^46^, ^47^, ^48^, ^49^, ^50^ Therefore, we limited our evaluation to the population more prone to social behavioral distortions, such as smoking and alcohol use, that is, the male population in Iran. Although our method and design may not be flawless, we hoped to achieve a better understanding of the possible risk and concurrent factors among this targeted population. Another factor was that ours was a single‐center study in a referral center in southwest Iran, which limited the study population diversity; however, there was no selection bias and the population was not skewed. We also included patients above 18 and below 60 years of age because there have been reports showing that the symptoms start before 35 years of age, while almost all report symptom onset before they are 50.^51^ However, other demographic data were not included in our study, which should be highlighted in future evaluations and studies. Also, detailed symptom evaluation was not performed while GERD was diagnosed based on the GERQ and patient symptoms, while the gold standard, ambulatory 24‐h pH monitoring, was not performed because of limited resources and the design of our study, especially to distinguish between GERD and FGIDs (reflux hypersensitivity and functional heartburn). Ultimately, further multicenter and population‐based studies are required to reach a final conclusion regarding the concurrence of these two diseases.

## Conclusion

Our results show that among male IBS patients, younger age, smoking, and alcohol use are among the risk factors for GERD. These findings may provide further insight into the best approach for treating these diseases; however, further multicenter international studies are required.

## Supporting information


**Figure S1**. Distribution of the incidence of gastrointestinal reflux disease (GERD) among male patients with inflammatory bowel syndrome (IBS).
