# Supplementary material for: Gastroesophageal reflux disease incidence among male patients with irritable bowel syndrome: A single‐center cross‐sectional study in southern Iran
Source: JGH Open. 2023 Jan 27;7(2):152–6. doi: 10.1002/jgh3.12867 (PMC9958336; doi:10.1002/jgh3.12867)
Supplement: Supplementary file 1 — Figure S1. Distribution of the incidence of gastrointestinal reflux disease (GERD) among male patients with inflammatory bowel syndrome (IBS). [file JGH3-7-152-s001.docx]

**Supplementary Figure 1.** Distribution of the incidence of gastrointestinal reflux disease (GERD) among male inflammatory bowel syndrome (IBS) patients.
